# Flow-S: A Field-Deployable Device with Minimal Hands-On Effort to Concentrate and Quantify *Schistosoma* Circulating Anodic Antigen (CAA) from Large Urine Volumes

**DOI:** 10.3390/diagnostics14080820

**Published:** 2024-04-16

**Authors:** Daniëlle de Jong, Cody Carrell, Jane K. Maganga, Loyce Mhango, Peter S. Shigella, Maddy Gill, Ryan Shogren, Brianna Mullins, Jay W. Warrick, John M. Changalucha, Govert J. van Dam, Khanh Pham, Jennifer A. Downs, Paul L. A. M. Corstjens

**Affiliations:** 1 Department of Cell and Chemical Biology, Leiden University Medical Center, 2300 RC Leiden, The Netherlands; 2 Salus Discovery LLC, Madison, WI 53703, USA; 3 Mwanza Intervention Trials Unit/National Institute for Medical Research, Mwanza, Tanzania; 4 Department of Parasitology, Leiden University Medical Center, 2300 RC Leiden, The Netherlands; 5 Division of Infectious Diseases, Weill Cornell Medicine, New York, NY 10065, USA; 6 Center for Global Health, Weill Cornell Medicine, New York, NY 10065, USA; 7 Department of Medicine, Weill Bugando School of Medicine, Mwanza, Tanzania

**Keywords:** circulating anodic antigen, CAA, equipment-free, field-deployable, lateral flow, LF, *Schistosoma*, point-of-care, POC, upconverting reporter particle, UCP, urine diagnostics

## Abstract

A laboratory-based lateral flow (LF) test that utilizes up-converting reporter particles (UCP) for ultrasensitive quantification of *Schistosoma* circulating anodic antigen (CAA) in urine is a well-accepted test to identify active infection. However, this UCP-LF CAA test requires sample pre-treatment steps not compatible with field applications. Flow, a new low-cost disposable, allows integration of large-volume pre-concentration of urine analytes and LF detection into a single field-deployable device. We assessed a prototype Flow-*Schistosoma* (Flow-S) device with an integrated UCP-LF CAA test strip, omitting all laboratory-based steps, to enable diagnosis of active *Schistosoma* infection in the field using urine. Flow-S is designed for large-volume (5–20 mL) urine, applying passive paper-based filtration and antibody-based CAA concentration. Samples tested for schistosome infection were collected from women of reproductive age living in a Tanzania region where *S. haematobium* infection is endemic. Fifteen negative and fifteen positive urine samples, selected based on CAA levels quantified in paired serum, were analyzed with the prototype Flow-S. The current Flow-S prototype, with an analytical lower detection limit of 1 pg CAA/mL, produced results correlated with the laboratory-based UCP-LF CAA test. Urine precipitates occurred in frozen banked samples and affected accurate quantification; however, this should not occur in fresh urine. Based on the findings of this study, Flow-S appears suitable to replace the urine pre-treatment required for the laboratory-based UCP-LF CAA test, thus allowing true field-based applications with fresh urine samples. The urine precipitates observed with frozen samples, though less important given the goal of testing fresh urines, warrant additional investigation to evaluate methods for mitigation. Flow-S devices permit testing of pooled urine samples with applications for population stratified testing. A field test with fresh urine samples, a further optimized Flow-S device, and larger statistical power has been scheduled.

## 1. Introduction

Schistosomiasis is a debilitating disease caused by infection with the helminth *Schistosoma*, with over 750 million people at risk and an estimated 250 million infections every year, of which the majority are in Africa [1]. Control strategies, such as preventive chemotherapy, mass drug administration (MDA) with the anti-schistosomal drug praziquantel (PZQ), improving access to clean water, sanitation, and hygiene, as well as snail control and community education, are being used in high *Schistosoma* endemic settings to reduce the burden of infection [2]. Central for successful and effective control plans is the use and implementation of accurate, cost-efficient, and user-friendly diagnostics.

The most common method to diagnose active infection in the field are still detection of parasite eggs in urine (*S. haematobium*) or stool (*S. mansoni* and *S. japonicum*), although these methods are labor-intensive and lack sensitivity [3], especially in low endemic settings. Egg excretion fluctuates on a daily basis [4] and depends on worm load (the number of healthy worm pairs), affecting detection of light infections.

A more sensitive and user-friendly laboratory-based test detects the *Schistosoma* circulating anodic antigen (CAA) in both serum and urine [5]. This CAA test is genus-specific, allowing detection of all *Schistosoma* species (including veterinary) in blood and urine (independent of the infecting species). It has the possibility to detect infections down to the presence of a single worm [6]. The test has a higher complexity, mainly relating to a sample treatment step that comprises extraction with trichloroacetic acid (TCA) followed by centrifugation. The resulting clear supernatant containing the targeted CAA (a carbohydrate structure) is then analyzed on a lateral flow (LF) test strip utilizing antibody capture in combination with a unique luminescent reporter, up-converting phosphor reporter particles (UCP). If necessary, higher (analytical) sensitivity can be achieved by employing ultrafiltration centrifugal columns to further concentrate the CAA in the TCA supernatant [5,7]. The ability to concentrate CAA from TCA extracted samples (urine in particular, as viscosity does not relevantly increase with concentration) did allow new pooling applications aimed at cost reduction as well as targeted sub-population based stratified testing to determine and monitor worm burden, as the level of CAA is directly related to worm burden [8]. The UCP-LF CAA test is well acknowledged and utilized in (basic) laboratories and numerous (multi-site) studies around the world [9,10,11], and is a recommended diagnostic in studies concerning schistosomiasis vaccine development [12,13]. Robust, user-friendly, dry-reagent formats of the test allow convenient worldwide shipping and storage at ambient temperature [14]. The UCP-LF CAA test applies a UCP label, which is a 400 nm submicron ceramic sphere composed of a silica coated ytterium oxysulfide (YOS) matrix doped with rare earth ions (ytterbium and erbium). This material is provided as a dry pellet in an assay well, but it is not optimal for full integration in the LF strip itself.

In the current study we explore the use of the Flow-S device as an alternative for the pretreatment of urine samples, and implement UCP-LF strips with a fully integrated 100 nm diamond shaped UCP with a sodium ytterium fluoride (YF) matrix. The ultimate goal is to develop an inexpensive disposable device that can be used with minimal hands-on effort for non-invasive diagnosis (using urine samples) for schistosome infections at the point-of-care and in the field with appropriate sensitivity and an acceptable (within 2 h) sample-to-result time.

## 2. Materials and Methods

### 2.1. Test Cohort and Clinical Samples

Ethical approval describing test protocols were provided by the National Institute for Medical Research in Dar es Salaam (NIMR/HQ/R8.a/Vol. IX/3625) and Weill Cornell Medicine in New York (20-10022745). Urine samples were collected from women of reproductive age living in a region of Tanzania in which *S. haematobium* infection is endemic. The women presented written informed consent and provided blood, urine, and stool samples to be tested for schistosome infection. Ten milliliters of urine was filtered and examined microscopically for *S. haematobium* ova in the field [15], and twenty milliliters of urine was frozen at −80 °C for the analysis with the Flow-S and UCP-LF CAA urine tests. The applied UCP-LF CAA urine test was a modification of the UCAA*hT*417 [5] format, here referred to as UCAA*hT*417-YF, as it utilized novel YF UCP nanomaterials suitable for dry integration in the LF strips for the Flow-S device. The equivalent of 417 μL urine sample was analyzed using the UCAA*hT*417-YF test format, which had a threshold of 2 pg/mL (Table 1). In short: 500 μL urine was extracted with 100 μL 12% *w/v* TCA, and upon centrifugation, 500 μL clear TCA-supernatant (equivalent of 417 μL urine) was concentrated to 20 μL using a 0.5 mL Amicon Ultra Centrifugal Filter device with 10 kDA molecular weight cutoff and used to run the UCP-LF CAA test strip; TCA extraction of urine (in contrast to serum) generally does not generate a significantly large precipitate. Serum CAA quantification was performed using the standard dry UCP assay (SCAA20, YOS submicron sized UCP), as previously described, with a CAA threshold of 30 pg/mL, utilizing the equivalent of 20 μL serum [16]. In short: 50 μL serum was extracted with 50 μL 4% (*w*/*v*) TCA, and upon centrifugation 20 μL clear TCA-supernatant was used to run the UCP-LF CAA test strip; the 20 μL TCA-supernatant was assumed to represent the extract of 20 μL serum after volume correction of the large pellet resulting from the TCA-extraction. Kato Katz testing [17] using five slides was performed on stool samples to rule out *S. mansoni* infection. Samples were classified as CAA positive or CAA negative based on the SCAA20 result: positive when CAA ≥30 pg/mL, negative when <30 pg/mL. A selection of urine samples from 15 serum positives (CAA ≥30 pg/mL) and 15 serum negatives (CAA < 30 pg/mL) were tested for presence of CAA in urine with Flow-S, and results were compared with the UCAA*hT*417-YF test.

### 2.2. Standard UCP-LF CAA Tests

UCP-LF sandwich assays depend on the use of a specific antibody pair to capture and to detect the targeted analyte. For CAA, a large repeating disaccharide (glycosaminoglycan-like structure), the same monoclonal antibody was used to capture and detect (sandwich) the antigen to both the Test (T) line on the LF strip and the UCP reporter. The Flow Control (FC) line on the LF strip captures UCP reporter particles that have flowed past the T-line; it indicates successful flow, and was used for normalization. Results are expressed as a Ratio value (R), which is the Test line signal (peak area, arbitrary units) divided by the Control line signal (peak area, arbitrary units). R values can be translated to CAA concentration using results obtained with CAA-spiked standard series.

Various formats of the UCP-LF CAA test are available. Table 1 shows the formats generally used indicating increased analytical sensitivity when including a concentration step with centrifugal Amicon devices with a molecular weight cutoff of 10 kDa. The SCAA500, which requires 500 μL serum or plasma, approaches the detection of a healthy single worm pair. CAA concentrations measured in urine are usually ≥10-fold less. With UCAAhT17 and UCAAhT417, CAA from the equivalent of 17 and 417 μL urine, respectively, was run over the LF strip. The UCAAhT417-YF introduced in this study utilized the UCP reporter particles with a YF ceramic matrix instead of a YOS ceramic matrix. YF particles are smaller, have a higher brightness per weight, and are more suited for dry integration in conjugate release pads of LF strips.

**UCP conjugation and dry conjugates:** Submicron-sized YOS:Yb^3+^, Er^3+^ (YOS; 400 nm) [18] and nano-sized NaYF_4_:Yb^3+^, Er^3^ UCP particles (YF: 110 × 80 nm; Intelligent Material Solutions Inc. Princeton, NJ, USA) [19] were coated with anti-CAA 147-3G4 antibody (LUMC, Parasitology) at a density of 25 μg antibody per mg UCP. Stock solutions (UCP at 1 mg/mL in storage buffer: 50 mM Glycine pH 8.0, 0.03% *v/v* Triton X-100, 0.1% *w/v* NaN_3_) were stored refrigerated at 4 °C until use [20].

SCAA20 assays were performed with the submicron-sized YOS:Yb^3+^, Er^3+^ coated UCP particles in a dry format using microtiter plate wells provided with 20 μL UCP-drying buffer (5% *w/v* sucrose, 0.5% BSA, 50 mM Tris pH 8, 135 mM NaCl and 0.25% Tween-20) containing 10 mg/mL YOS UCP (200 ng UCP/well) and dried overnight at 37 °C [14].

For the UCAA*hT*417-YF assay, dried nano-sized YF particles for 100 assays were produced by adding 20 μL UCP-drying buffer containing 5 mg/mL UCP (10 μg UCP, 100 ng UCP per LF test strip) in an Eppendorf tube, and dried overnight at 37 °C.

UCP-LF test strips for Flow-S with nano-sized YF particles integrated in an untreated conjugate release pad were produced by spraying 1 μL UCP-drying buffer containing 0.5 mg/mL UCP per 5 mm width (500 ng UCP per LF strip).

**UCP-LF CAA test strips for the SCAA20 and UCAA*hT*417-YF assays:** LF strips (4 mm width, 5 cm length) were produced as described earlier [20]. In brief, a 2.5 cm nitrocellulose membrane (Sartorius UniSart CN95) comprising a Test line with monoclonal mouse anti-CAA antibody (250 ng/strip; 147-3G4, LUMC, Parasitology) and Flow Control line with a polyclonal goat anti-mouse antibody (100 ng/strip; M8642, Sigma-Aldrich, St. Louis, MO, USA) was positioned on a self-adhesive backing card (KN-V1050R; Kenosha, Amstelveen, The Netherlands) together with a 1 cm glass fiber sample pad (Kenosha; #8964) on the front end and a 2 cm absorbent pad (Whatman #470; Kenosha) on the back end.

**SCAA20 assay (dry format; threshold 30 pg/mL):** Banked serum samples and standard series (CAA spiked in negative serum of non-endemic origin, with a test value well below the threshold or zero) were thawed at room temperature; 50 μL was extracted with one volume of 4% (*w*/*v*) TCA (Trichloroacetic acid; Merck 1.00807) and centrifuged. Of the clear supernatant, 20 μL was added to 50 μL high salt LF buffer (HSLF: 200 mM Tris pH 8.0, 270 mM NaCl, 1% *w/v* BSA, 0.5% *v/v* Tween-20) and added to microtiter plate wells containing 50 μL HSLF with 200 ng hydrated YOS-UCP conjugate. After incubation for 1 h in a thermoshaker (37 °C, 900 rpm), a UCP-LF CAA test strip was added to initiate LF [20].

**UCAA*hT*417-YF assay (threshold 2 pg/mL):** Banked urine samples and CAA standard series (spiked in CAA negative urine of non-endemic origin, with a test value well below the threshold or zero) were thawed at room temperature; 0.5 mL was extracted with 0.1 mL 12% TCA and centrifuged, and 0.5 mL of clear supernatant was concentrated to 20 μL utilizing a 10 kDa cutoff Amicon Ultra-0.5 Centrifugal Filter device (UFC5010; Merck, Darmstadt, Germany). The concentrate was mixed with 100 μL HSLF buffer containing 50 ng hydrated YF-UCP conjugate, pipetted in a microtiter well, and a UCP-LF CAA test strip was added to initiate LF.

### 2.3. UCP-LF CAA Test in the Flow-S Device

**Figure 1:**
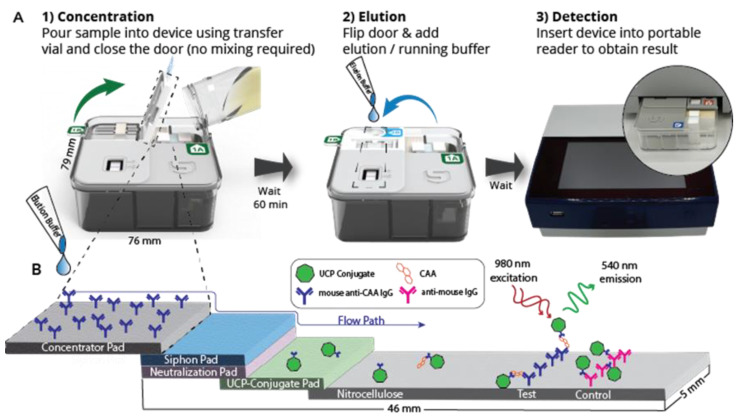
Flow-S device and test protocol. (**A**) The Flow-S workflow. (1) Concentration. The workflow for Flow-S begins by adding a urine sample to a transfer vial (typically after collecting the urine in a standard urine cup). The transfer vial contains a buffer to ensure the urine is at the proper pH and uses a “fill-to” line to ensure the correct volume is added. Urine is then poured from the transfer vial into the Flow device. After the urine is added, a door containing the concentrator pad with immobilized anti-CAA antibodies is closed. The entire sample will flow across the capture pad and interact with the capture antibodies over a period of 1 h, and the CAA from the 10 mL urine sample will be concentrated onto the pad. (2) Elution. The concentrator pad is flipped to the other half of the device where it interfaces with the UCP-LF CAA test strip. A low-pH elution buffer is added to the concentrator pad to elute CAA from the capture antibodies. After elution, the CAA flows through a neutralization pad to increase the pH to ~7.5 before reaching the UCP-LF CAA test strip. Here, the UCP conjugate binds eluted CAA before the complex is captured at the test line. A “siphon pad” (with proprietary configuration, [21]) upstream of the neutralization pad physically wicks and sequesters the precise volume of surplus urine remaining in the concentrator pad after concentration away from the primary flow path. This siphon pad prevents this remaining urine from passing onto the neutralization pad of the UCP LFA and interacting/interfering with detection reagents, yet allows the subsequent volume of elution buffer containing analyte to flow onto the LFA for detection. (3) Detection. After 30 min the UCP-LFA can be read using a portable, battery-powered reader with an IR excitation source. (**B**) The mechanism of the UCP excitation is critical to the performance of the LF. Here the UCPs are excited at 980 nm and emit at 540 nm. The anti-stokes shift of the UCPs greatly improves signal to noise ratio over traditional fluorescent reporters.

**Flow-S UCP-LF CAA test strips:** LF strips (5 mm width) for integration into the Flow-S device were provided with a conjugate release pad containing 500 ng of dry YF-UCP conjugate (identical to the UCP conjugate used for UCAA*hT*417-YF), and an additional neutralization pad (1 M Tris pH8, 1.5% Casein and 1% sucrose) upstream of the conjugate release pad, separated by an untreated, absorbent “siphon pad” (see Figure 1 image and caption for details).

**The Flow-S assay:** Urine samples were thawed at room temperature and mixed thoroughly to homogenize precipitates before adding 10 mL to a transfer vial that contained a buffer to ensure the urine was at a neutral pH. The vial uses a “fill-to” line to ensure the correct volume is added. Urine was then poured into the Flow-S device to initiate filtration and subsequent antibody based binding of CAA to the Flow-S capture pad containing monoclonal mouse anti-CAA antibody (147-3G4); urine filtration and CAA concentration was allowed to continue for 60 min. Subsequently, the Flow-S door (containing the capture pad with captured CAA, Figure 1) was flipped to achieve direct contact with the integrated UCP-LF CAA test strip. To release CAA from the capture pad and to initiate LF, 225 μL of pH 2 elution buffer (Pierce IgG Elution Buffer, pH 2.0; 21004, Thermo Fisher, Waltham, MA, USA) supplemented with 0.5% Tween-20 (*v*/*v*), and 270 mM NaCl was added [21].

### 2.4. CAA Quantification and LF Strip Analysis

**CAA standard series:** A stock solution of standard CAA (TCA-soluble fraction of *Schistosoma* adult worm antigen containing 3% *w*/*w* CAA) [20] was used to prepare appropriate standard series. Standard series of CAA from 1000, 316, 100, 31.6, 10, 3.16, 1, and 0 pg/mL were spiked in banked (frozen) CAA-negative serum and CAA-negative urine. Both of these negative controls were of non-endemic origin, with a test value well below the threshold or 0. Urine CAA standards above 100 pg/mL were not tested with the Flow-S device because of limited amounts of CAA standard.

**UCP strip readers:** The SCAA20 assay has been implemented at the NIMR laboratory in Mwanza and used in multiple studies as a means to identify infection [22,23,24,25]. LF strips are analyzed using a benchtop UPCON reader suited for UCP (Labrox Oy, Turku, Finland) with the ability to read up to 20 LF strips at a time. LF strips from the UCAAhT417-YF were analyzed on the UPCON reader as well as a portable reader custom adapted for UCP applications (UCP-Quant, version LR3; DIALUNOX, Stockach, Germany). The Flow-S device is compatible with the UCP-Quant reader, adapted such that the LF strips are able to be read without removing them from the Flow-S device. UCP applications require IR excitation (980 nm), and for the UCP materials used here, detection of green emission light (phosphorescence) at 540 nm. Independent of the reader, results can be expressed as a Ratio value (R), which is the Test line signal (peak area, arbitrary units) divided by the Control line signal (peak area, arbitrary units). R values can be translated to CAA concentration using results obtained with CAA standard series [26].

## 3. Results

### 3.1. Description of the Flow-S Device

Flow is a fully integrated disposable test device that only requires addition of the urine sample into the device for pre-concentration, and addition of an elution/running buffer to initiate LF detection. The Flow device is compatible with an adapted portable/standalone reader (UCP-Quant LR3, adapted for the UCP reporter technology), such that LF strips do not need to be removed from the Flow device for analysis. Figure 1 depicts the device in a schematic indicating the procedural steps. While Flow is described here specifically for CAA detection in urine (i.e., Flow-S), the Flow device is a modular test platform that can be scaled for different volumes and allows rapid integration of capture antibodies and LF strips for other targets, with only minor modifications.

Further, Flow can also leverage other, non-antibody-based biomarker capture/recognition technologies. The biomarker-specific components of the fully integrated device include the interchangeable concentrator pad and the LF strip. The Flow-S application described in this study evaluates the potential to detect *Schistosoma* derived CAA (a carbohydrate) in the urine of the host, the presence of which indicates an ongoing active parasitic worm infection.

### 3.2. Targeted Sensitivity of the Flow-S Device

**Figure 2:**
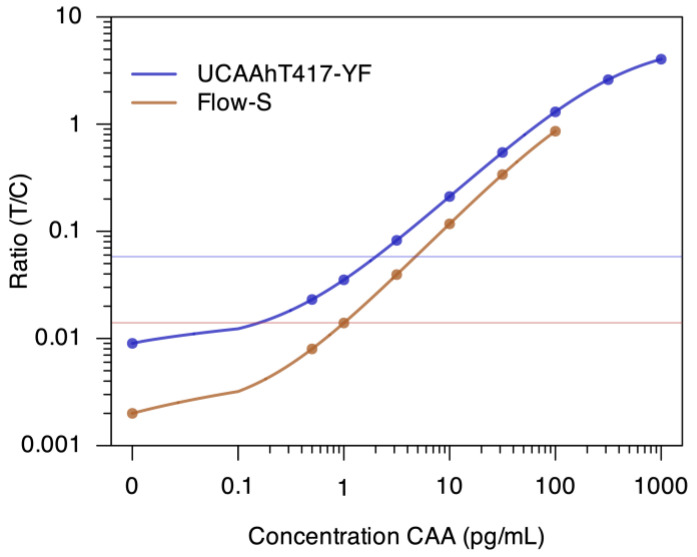
Curve fit of CAA standard series in urine and LLOD of both Flow-S and UCAAhT417-YF. Figure 2 shows curve fits (4-parameter logistic fit; logical scaling with transition from linear to logarithmic at 0.1 pg/mL) [27] of a representative test with a CAA standard series spiked in CAA-negative urine: UCAAhT417-YF with the equivalent of 417 μL urine after TCA extraction and concentration by ultracentrifugation, Flow-S with 10 mL untreated urine. Results are given as (log) Ratio value (Test line divided by Flow Control line) versus (log) CAA concentration. Ratio values obtained with negative samples and repetitive testing indicated an approximated LLOD of 2 pg/mL (R = 0.061) for the UCAAhT417-YF dry assay format. For the Flow-S an LLOD of 1 pg/mL (R = 0.014) was achieved with a Pearson correlation coefficient for the standard series of 0.983 as compared to the UCAAhT417-YF. Optimization of both assays to demonstrate feasibility was limited. The dynamic range was three orders of magnitude or better, approaching a maximum plateau value for the UCAAhT417-YF above 1000 pg/mL; samples with values >1000 pg/mL would require retesting with a smaller volume to determine an accurate CAA concentration. For the Flow-S device, the highest concentration tested was 100 pg/mL because of the large sample volume (10 mL) and limited availability (cost) of CAA standard.

To implement the UCP reporter as a dry reagent in the conjugate release pad of the Flow-S LF strip, nano-sized YF reporter particles were applied, rather than the submicron-sized YOS reporter particles used in the SCAA20 serum based assay (Table 1). With this first prototype of Flow-S utilizing 10 mL urine, a LLOD of 1 pg CAA/mL was targeted. Dry format YF particles were also used for the laboratory-based UCAAhT417-YF assay, although not integrated as part of the LF strip but as a dry pellet in a microfuge tube requiring hydration and subsequent mixing with the sample prior to initiating LF. The UCAAhT417-YF test materials were produced in-house at LUMC and demonstrated a lower limit of detection threshold of 2 pg CAA/mL (Figure 2).

### 3.3. Evaluation of the Flow-S Device with Banked Urine Samples

Serum and urine samples were collected in a region of Tanzania in which *S. haematobium* infection is endemic. All stool and urine samples were analyzed for the presence of eggs. As expected, analysis of stool samples did not indicate the presence of *S. mansoni* eggs. Urine filtration and microscopic examination of the filtrate indicated four samples with *S. haematobium* eggs.

The egg-positive urine samples also tested positive for CAA in serum (SCAA20 assay), with CAA serum concentrations ranging from 37.8 to 641 pg/mL. With CAA concentrations on average being 10-fold lower in urine [28], a group of 15 negatives (CAA serum < 30 pg/mL) and 15 positives (CAA serum ≥ 30 pg/mL, including the 4 egg positives) in the anticipated CAA urine range of 1–1000 pg/mL were chosen for testing (banked) untreated urine with the Flow-S device in comparison to the UCAAhT417-YF assay with required sample treatment.

Table 2 shows the results, with samples ranked by SCAA result, which is the CAA concentration in serum: the urine samples were tested single-blind. Samples with serum CAA values <30 pg/mL were assigned to the CAA negative class, but potentially may be ‘false’ negatives as some participants were included who had previously tested positive and may have remained low-positive after their praziquantel (PZQ) treatment; for this feasibility study, linking with other patient records was not essential. Results with the UCAAhT417-YF matched with serum CAA positive results, except for one sample (#14) with a CAA serum concentration of 86 pg/mL, which was not identified as CAA positive with the urine assay. In contrast, two samples that were read as serum CAA negative (#22, 23) did score positive with the UCAAhT417-YF, but with only 3.2 and 4.4 pg/mL, respectively, which are close to the assay cutoff threshold. Validation of the positive urine test result with a larger sample volume [5] was not performed.

**Figure 3:**
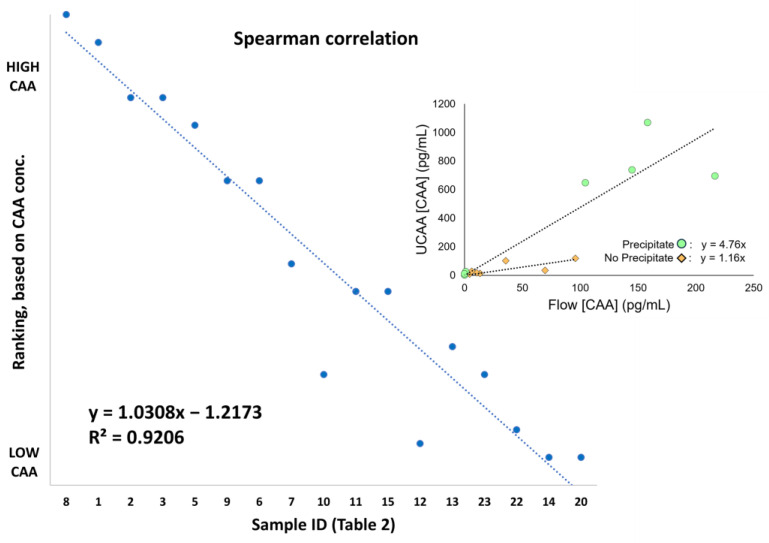
Spearman correlation between Flow-S and UCAAhT417-YF and CAA concentrations measured by the UCAAhT417-YF (UCAA) assay vs. Flow-S assay stratified by presence of sediment.

Results obtained with the Flow-S device correlated well with the UCAAhT417-YF. From the 14 SCAA20 positive samples identified with the UCAAhT417-YF, the Flow-S device identified 12. Of the two samples missed, one (#12) had a low urine CAA concentration, 11.4 pg/mL, as determined with the UCAAhT417-YF. For the other sample (#4), an egg-positive sample with high serum CAA concentration and 110 pg/mL in urine as determined with the UCAAhT417-YF, the Flow-S result was deemed invalid because insufficient volume flowed through the device (only around 10% of the input) due to clogging. Clogging may have occurred because of precipitates, although this was excessive, in combination with other so far undefined factors. Flow-S also scored three (#20, 22, 23) serum negative samples as positive, but all three samples were just above the threshold of 1 pg/mL, with values of 1.5, 1.0, and 1.7 pg/mL. Two of these samples also scored above the positivity threshold with the UCAAhT417-YF. Figure 3 shows the Spearman ranking of the 17 samples based on determined urine CAA concentration above the threshold with either of the two urine assays (Table 2). Samples #10 and #12, with the UCAAhT417-YF (*x*-axis) deviating the most, were observed to have the largest precipitate. A conceivable correlation of (freeze-thawing) precipitate to signal reduction could be visualized by plotting UCAAhT417-YF and Flow-S signals of samples from the SCAA20 positive group with and without noted precipitate (Figure 3, inlay). Samples without precipitate had a regression slope of 1.16, indicating overall agreement between the assays for these samples (ideal value = 1). In contrast, samples with precipitate had a slope of 4.76, indicating a four to five-fold reduced signal in the Flow-S assay relative to the UCAAhT417-YF assay for these samples. It was also observed that the four highest levels of infection were associated with precipitate, which may include cell debris (e.g., due to potential hematuria associated with infection). Thus, the presence of precipitates seems to lead to larger differences in signals (see also Table 2; Conc UCAA/Flow Factor). Although the expectation is that precipitates mainly occurred as a consequence of storage and are absent in fresh urines, the composition, amount, and potential occurrence in fresh urines may require further investigation to establish an ideal maximum time between collection and testing.

While low sample numbers and the use of a non-random selection based on serum results prohibit properly powered evaluation of sensitivity and specificity, the performance of Flow-S in this study indicates that it has good potential to achieve WHO targets as a point-of-care schistosomiasis diagnostic. The main focus of such a diagnostic, as specified by WHO diagnostic ideal target profiles (TPP), should be a high specificity of 98.5% with a sensitivity of 78%, whereas minimum conditions allow 2 h to develop the test result, which should then remain stable for at least 30 min [29]. When using the results of the serum CAA assay as the reference standard and a positivity threshold for the Flow-S of 1 pg/mL, there was a 92.8% rate of positive percent agreement (PPA), with 80% negative percent agreement (NPA) (implying 13 true positives, 3 false positives, 12 true negatives, and 1 false negative). Increasing the Flow-S positivity threshold to 2 pg/mL would result in 78.6% PPA and 100% NPA (implying 12 true positives, 0 false positives, 15 true negatives, and 3 false negative).

**Figure 4:**
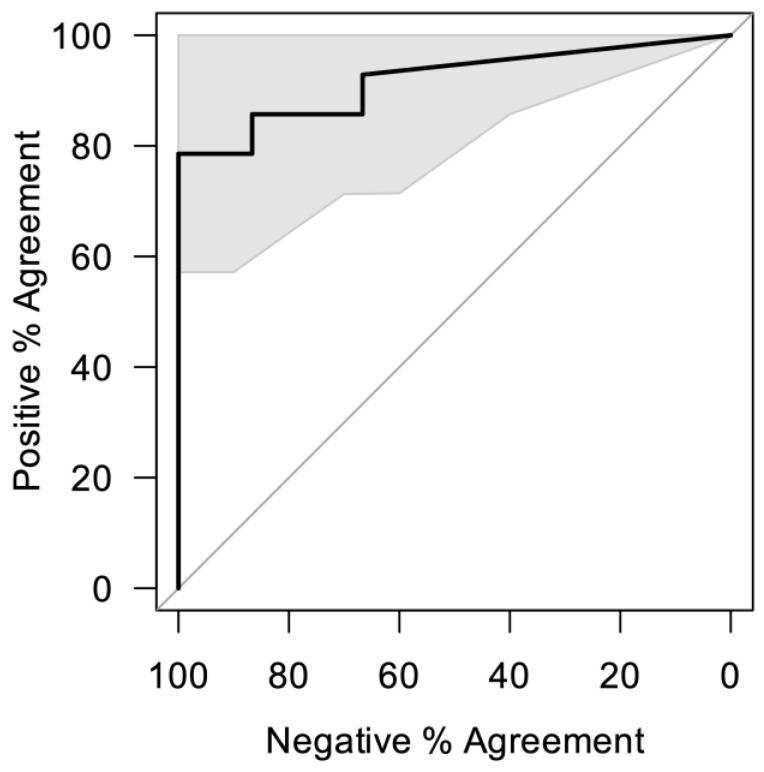
Flow-S receiver operating characteristic (ROC) curve using SCAA results as the comparator or reference assay.

A 1 pg/mL threshold was chosen instead of 2 pg/mL because of the analytical sensitivity of the assay determined in the laboratory across many (>10) true negative urine samples. The dependence of clinical performance on the threshold is demonstrated by the ROC curve in Figure 4. The threshold may be adjusted for different use cases requiring different performance characteristics (e.g., prevalence testing for MDA vs. individual test and treat). Thus, the “best” threshold for the urine assays depends upon the application, as evidenced by different WHO recommendations for multiple use cases [29]. Furthermore, in very low intensity infections, the reference SCAA assay may be incorrect, and small deviations at low analyte concentrations can lead to imperfect correlations between urine and serum CAA. For example, the Flow-S and UCAA assays agree with respect to one false-negative and two false-positives, yet due to the nature of the analysis such disagreements with the serum results negatively impact the PPA and NPA of Flow-S and UCAA. However, serum CAA remains the most well-established reference standard, and the results were in close agreement.

## 4. Discussion

This study evaluated CAA testing using large-volume urine samples with a novel, fully integrated test device (Flow-S) with minimal hands-on effort/time (<1 min), aiming to remove the need for sample treatment procedures that require a basic laboratory setting. The test device consisted of a passive filtration-based antigen concentration segment utilizing antibody capture in combination with fully integrated quantitative UCP-LF CAA test strips. The latter required implementation of dry anti-CAA UCP reporter-conjugate on the LF strip; nano-sized YF UCP reporter particles were applied instead of the previously used reporter conjugates with sub-micron UCP YOS particles, as these were not ideal for dry integration in the LF strip.

Here, we compared a field-applicable user-friendly prototype Flow-S device utilizing 10 mL untreated urine to a laboratory-based UCP-LF CAA assay format analyzing 417 μL of pre-treated urine requiring TCA treatment and centrifugation. The concurrent implementation and use of dry nano-sized YF reporter particles was performed according to protocols initially evaluated and applied for the detection of cellular response markers (e.g., cytokines) in serum [30], however without methodical optimization for urine-based *Schistosoma* CAA detection within the Flow-S and UCAAhT417-YF test formats.

The Flow-S device allows direct testing of large-volume urines (up to 20 mL) without prior sample preparation, and the potential of Flow-S for test applications outside the laboratory in field settings is obvious. The detection limit indicated similar analytical sensitivity in comparison to the laboratory based UCAAhT417-YF test, although a larger sample volume is required for the Flow-S device. Because collection of urine is non-invasive, the need for a larger urine volume is not a concern or limitation. The current Flow-S device can detect CAA spiked into 10 mL of urine at 1 pg/mL. Further optimization is required to achieve the sensitivity needed to identify ultralow infection grades. In serum, CAA levels between 1 and 10 pg/L were thought to represent an infection with a patent single worm pair [31]. Lower levels from single sex infections [6] and worms recovering after PZQ treatment are possible. In urine, CAA levels may be >10-fold lower than in serum; this difference will vary per individual and depend on fluid intake and the time of urine sample collection. Additionally, the formation of precipitates in urine upon storage may further deplete soluble CAA that can be assessed with the Flow-S device. Utilizing freshly collected urines, the LLOD target for a future optimized Flow-S device should therefore be 0.1 pg/mL or better. The current 1 pg/mL level for urine allowed detection of individuals with serum CAA levels well below 100 pg/mL, designated low-to-medium infections, often undetectable by parasitological methods (egg count). The unoptimized Flow-S device with LLOD of 1 pg CAA/mL easily outperformed identification of *S. haematobium* infection by egg count.

The modular Flow device used in this study is a generic test platform theoretically suited for all biomarkers present in urine that can be captured and detected by an antibody; biomarker specific components only relate to the replaceable concentrator pad and LF detection test strip. For the Flow-S device targeting *Schistosoma* CAA, alternative capture methods compatible with the highly negatively charged CAA molecule can be considered. For example, the use of positively charged poly(amidoamine) (PAMAM) dendrimers would not require acid elution of CAA from the concentrator pad [32]. Use of different capture technologies will require some modification of the (dry) reagents. The Flow system was originally developed by Salus Discovery for detection of mycobacterial lipoarabinomannan (LAM) antigen in urine as a point-of-care test for tuberculosis with visual detection [33].

In the current study, the Flow system was successfully combined with a luminescent high sensitivity label, up-converting reporter materials. Additionally, the Flow-S device was evaluated with 10 mL of banked frozen urines, rather than fresh urines. The device clearly outperformed the conventional parasitological approach of microscopic egg counts performed on a urine filtrate of 10 mL. In comparison to the laboratory based UCAAhT417-YF test (besides one failure), only 1 out of the 13 serum positives was missed with Flow-S. That particular urine sample had an obvious precipitate and also scored at the lower end of the UCAAhT417-YF curve with a concentration of 11.4 pg/mL. The presence of precipitates affects accurate determination of quantified CAA concentration and is one of the reasons that a sample preparation step (i.e., the TCA extraction) is included for the laboratory-based UCAA test formats. The formation of excess precipitates is mainly a problem when storing and freeze-thawing urine samples [34]. Future prospective field experiments with fresh urines will clarify the influence on the measured CAA concentration of potential precipitates in fresh urines. So far, instant determination of CAA levels using the UCP-LF CAA laboratory based method on freshly collected urines has never been performed. Testing of urine samples stored for three weeks at ambient temperature did not indicate a decrease in CAA levels, although cloudiness and obvious biochemical or microbiological changes had occurred [7]. However, TCA treatment (included in the sample pre-treatment step of UCP-LF CAA test) to retain CAA in solution was crucial, as it dealt with precipitates that would otherwise capture CAA from solution.

On average, Flow-S returned CAA concentrations which were four to five-fold lower than those determined with the UCAAhT417-YF test in samples with notable precipitate. Urine samples for which both tests returned a CAA concentration in the same range were noted to be free of precipitates. Flow-S testing with freshly collected urines is expected to demonstrate quantitative results more closely approximating the UCAAhT417-YF test results. There may be a correlation between precipitate and degree of infection, as observed in the samples with the four highest levels of CAA. High specificity of the UCP-LF and Flow-S assays is likely aided by preventing interaction of many urine components with the LF strip itself. In the case of the UCP-LF assays, TCA extraction, centrifugation to remove precipitate, and high-molecular weight filtration largely prevent urine protein and potential cell debris from interacting with the LF strip. In the case of the Flow-S assay, the urine sample passes through the concentration pad and is sequestered within absorbent pads of the device body, and then the capture pad is moved to the LF test where the CAA is eluted with clean elution buffer, minimizing interaction of urine with the LFA test components.

This study was limited by the number of samples and non-random selection. Valid clinical sensitivity and specificity results cannot be extracted from this small cohort. As a first pilot to evaluate the potential of the Flow-S device to detect and quantify CAA from untreated urines, the outcome was definitely successful. Similar performance was observed in comparison to an adapted urine UCP-LF CAA test (UCAAhT417-YF), which required a demanding (not field-applicable) sample pre-treatment step. Comparison to serum-CAA levels, as determined with a worldwide-applied and well-acknowledged UCP-LF CAA test, supports the excellent performance of Flow-S, which is expected to meet the WHO TPP in terms of clinical sensitivity and specificity, as well as other indicated performance factors. Moreover, as Flow-S performs well for this *S. haematobium* cohort, it can be expected to perform even better for *S. mansoni*, given the higher CAA concentrations observed for the latter species [35]. Appraisal of the Flow-S for field usability was tested in the laboratory by technicians focused on omitting laboratory-requiring tools, and it successfully passed in terms of user-friendliness.

Anticipated advancements in the Flow-S device envisage an improvement of the LLOD to the 0.1 pg CAA/mL level or better. This level will allow identification of low worm infection grades. It would also make the Flow-S device usable to analyze pooled urine samples in field settings for stratified testing, targeting high risk groups to assist with real-time decisions regarding the need for mass drug treatment.

## Figures and Tables

**Table 1:** UCP-LF CAA assay formats: lower limit of detection.

Assay Format *	Amicon Device ^†^	Threshold (pg/mL) ^§^ Dry vs. Wet
SCAA20	None	30 vs. 10
SCAA500	0.5 mL	3 vs. 1
UCAA*hT*17	None	20 vs. 6
UCAA*hT*417	0.5 mL	2 vs. 0.6
UCAA*hT*417-YF	0.5 mL	2 vs. 0.6

Table showing quality control (QC) guaranteed lower limit of detection (LLOD) of UCP-LF test materials utilizing sub-micron YOS reporter particles, as determined by standard series in appropriate biological matrix. * The sample matrix is indicated by ‘S’ for serum and ‘U’ for urine. The indication ‘hT’ specifies the use of higher trichloroacetic acid (TCA) concentration for extraction: urine sample extracted with ⅙ volume of 12% *w/v* TCA rather than 1 volume of 4% TCA as used for serum; final concentration of the TCA supernatant is always 2% *w/v* TCA. The number indicates the equivalent of the volume of the original clinical sample analyzed on the UCP-LF CAA test strip in μL. TCA extracted urine samples, in contrast to TCA extracted serum, can be further concentrated without fluidity (viscosity) issues becoming a relevant factor in the LF analysis. The indication YF specifies the use of nano-sized UCP reporter particles in a YF matrix, rather than submicron-sized YOS particles under non-optimized assay conditions. ^†^ Amicon centrifugal devices with a 10 kDa molecular weight cutoff. ^§^ QC threshold for the assay using dry (hydrated air-dried pellet) versus wet (freshly sonicated liquid) UCP conjugate, respectively.

**Table 2:** Evaluation samples ranked on CAA serum concentration as determined with the SCAA20 assay.

	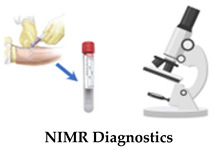			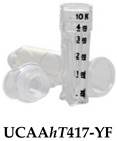			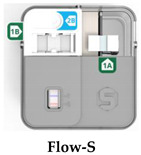				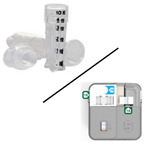
ID	Class	SCAA20 pg/mL	Eggs 10 mL	Ratio T/C	Conc pg/mL	SCAA/ UCAA Factor	Ratio T/C	Conc pg/mL	Precip.	SCAA/Flow Factor	UCAA/Flow Factor
1	Positive	37,750	13	3.560	845.6	44.6	1.164	158.3	S	238.5	5.3
2	Positive	9915	0	3.300	682.5	14.5	0.886	104.5	S	94.9	6.5
3	Positive	7733	0	2.864	482.0	16.0	1.101	144.8	M	53.4	3.3
4	Positive	4848	69	1.225	110.1	44.0	invalid	invalid	S	na	na
5	Positive	4767	0	1.421	137.2	34.8	0.833	95.8		49.8	1.4
6	Positive	1872	0	0.539	36.5	51.3	0.654	69.5		26.9	0.5
7	Positive	1168	0	0.467	30.4	38.4	0.074	6.2		188.8	4.9
8	Positive	686	75	3.676	933.3	0.7	1.394	216.6	S	3.2	4.3
9	Positive	642	24	1.112	95.9	6.7	0.375	35.5		18.1	2.7
10	Positive	295	0	0.408	25.6	11.5	0.015	1.1	M	267.0	23.2
11	Positive	276	0	0.357	21.6	12.7	0.123	10.5		26.2	2.1
12	Positive	200	0	0.214	11.4	17.6	<0.014	<1	L	na	na
13	Positive	92	0	0.206	10.8	8.5	0.052	4.2		21.8	2.6
14	Positive	86	0	<0.061	<2	na	<0.014	<1	S	na	na
15	Positive	72	0	0.262	14.7	4.9	0.154	13.3		5.4	1.7
16	Negative	<30	0	<0.061	<2	na	<0.014	<1	S	na	na
17	Negative	<30	0	<0.061	<2	na	<0.014	<1		na	na
18	Negative	<30	0	<0.061	<2	na	<0.014	<1	S	na	na
19	Negative	<30	0	<0.061	<2	na	<0.014	<1		na	na
20	Negative	<30	0	<0.061	<2	na	0.019	1.5		na	na
21	Negative	<30	0	<0.061	<2	na	<0.014	<1		na	na
22	Negative	<30	0	0.082	3.2	na	0.014	1.0	S	na	3.2
23	Negative	<30	0	0.103	4.4	na	0.022	1.7		na	2.6
24	Negative	<30	0	<0.061	<2	na	<0.014	<1		na	na
25	Negative	<30	0	<0.061	<2	na	<0.014	<1	S	na	na
26	Negative	<30	0	<0.061	<2	na	<0.014	<1	S	na	na
27	Negative	<30	0	<0.061	<2	na	<0.014	<1	S	na	na
28	Negative	<30	0	<0.061	<2	na	<0.014	<1		na	na
29	Negative	<30	0	<0.061	<2	na	<0.014	<1		na	na
30	Negative	<30	0	<0.061	<2	na	<0.014	<1	M	na	na

Table evaluating samples ranked on CAA serum concentration as determined with the SCAA20 assay. Urine CAA levels were determined from paired urine samples with the UCAAhT417-YF and Flow-S device; both Ratio values (R = T/C) and CAA concentrations are shown. Approximated LLOD for both assays: UCAAhT417-YF, R ≥ 0.061 relates to 2 pg CAA/mL; Flow-S, R ≥ 0.014 relates to 1 pg CAA/mL. Samples that contained precipitates after thawing were marked S for low, M for medium, and L for excessive sediment. A factor indicating the difference in concentration between UCAAhT417-YF and Flow-S is indicated for CAA-positive samples.

## Data Availability

The raw data supporting the conclusions of this article will be made available by the authors on request.
